# Antitrypanosomal 8-Hydroxy-Naphthyridines Are Chelators of Divalent Transition Metals

**DOI:** 10.1128/AAC.00235-18

**Published:** 2018-07-27

**Authors:** Richard J. Wall, Sonia Moniz, Michael G. Thomas, Suzanne Norval, Eun-Jung Ko, Maria Marco, Timothy J. Miles, Ian H. Gilbert, David Horn, Alan H. Fairlamb, Susan Wyllie

**Affiliations:** a Wellcome Trust Centre for Anti-Infectives Research, Division of Biological Chemistry and Drug Discovery, School of Life Sciences, University of Dundee, Dundee, United Kingdom; b Diseases of the Developing World, GlaxoSmithKline, Madrid, Spain

**Keywords:** chelator, drug discovery, kinetoplastids, mechanisms of action, transition metals

## Abstract

The lack of information regarding the mechanisms of action (MoA) or specific molecular targets of phenotypically active compounds can prove a barrier to their development as chemotherapeutic agents. Here, we report the results of our orthogonal genetic, molecular, and biochemical studies to determine the MoA of a novel 7-substituted 8-hydroxy-1,6-naphthyridine (8-HNT) series that displays promising activity against Trypanosoma brucei and Leishmania donovani. High-throughput loss-of-function genetic screens in T. brucei highlighted two probable zinc transporters associated with resistance to these compounds. These transporters localized to the parasite Golgi apparatus. Directed by these findings, the role of zinc and other divalent cations in the MoA of these compounds was investigated. 8-HNT compounds were found to directly deplete intracellular levels of Zn^2+^, while the addition of exogenous Zn^2+^ and Fe^2+^ reduced the potency of compounds from this series. Detailed biochemical analyses confirmed that 8-HNT compounds bind directly to a number of divalent cations, predominantly Zn^2+^, Fe^2+^, and Cu^2+^, forming 2:1 complexes with one of these cations. Collectively, our studies demonstrate transition metal depletion, due to chelation, as the MoA of the 8-HNT series of compounds. Strategies to improve the selectivity of 8-HNT compounds are discussed.

## Abstract (precis)

The lack of information regarding the mechanisms of action (MoA) or specific molecular targets of phenotypically active compounds can prove a barrier to their development as chemotherapeutic agents. Here, we report the results of our orthogonal genetic, molecular, and biochemical studies to determine the MoA of a novel 7-substituted 8-hydroxy-1,6-naphthyridine (8-HNT) series that displays promising activity against Trypanosoma brucei and Leishmania donovani.

## INTRODUCTION

The protozoan parasites Trypanosoma brucei, Trypanosoma cruzi, and Leishmania spp. are the causative agents of the human infections sleeping sickness, Chagas' disease, and leishmaniasis, respectively. These diseases are responsible for more than 74,000 fatalities annually and the loss of over 4,600,000 disease-adjusted life years (1). Some of the poorest areas of the world are afflicted by these vector-borne parasites, and the accompanying economic burden provides a major obstacle to improving human health (2). There are no approved therapeutic vaccines, and current treatments for protozoan diseases suffer from a range of problems, including severe toxicity and emerging drug resistance (3–5). To compound these difficulties, many of the current chemotherapeutic treatments require lengthy periods of hospitalization and are prohibitively expensive (2). Thus, there is an urgent need for better, safer, efficacious drugs that are fit for purpose in resource-poor settings.

Antitrypanosomatid drug discovery has been hindered by a paucity of robustly validated drug targets in these parasites (6). This has severely limited target-focused screening programs and has increased reliance upon phenotypic screening of parasites to identify start points for drug discovery. Phenotypic approaches, involving the screening of compounds directly against intact parasites in *in vitro* culture, have proven effective (7). However, a lack of information regarding the mechanism(s) of action (MoA) or specific molecular target(s) of these active compounds can often prove a barrier to their optimization in order to overcome pharmacokinetic and/or toxicity issues. A more complete understanding of MoA can also facilitate the prioritization of compounds with novel and promising targets or the deprioritization of compounds with known unattractive or failed targets, such as sterol 14α-demethylase (CYP51) in T. cruzi (8, 9). Furthermore, such knowledge can inform future drug combination strategies and may identify novel mechanisms that could be exploited *de novo* for target-based drug discovery.

Here, we describe orthogonal genetic, molecular, and biochemical studies focused on determining the MoA of a novel 7-substituted 8-hydroxy-1,6-naphthyridine (8-HNT) series that displayed promising activity against T. brucei and Leishmania donovani. This compound series emerged from screening a 1.8-million-compound library against L. donovani as part of a collaboration between GlaxoSmithKline (GSK) and the University of Dundee Drug Discovery Unit (10). Our comprehensive MoA studies reveal that 8-HNT compounds chelate transition metals and that this chelation forms the basis of their cytotoxicity in protozoan parasites.

## RESULTS

### Identification and development of an antitrypanosomal 8-HNT series.

**FIG 1:**
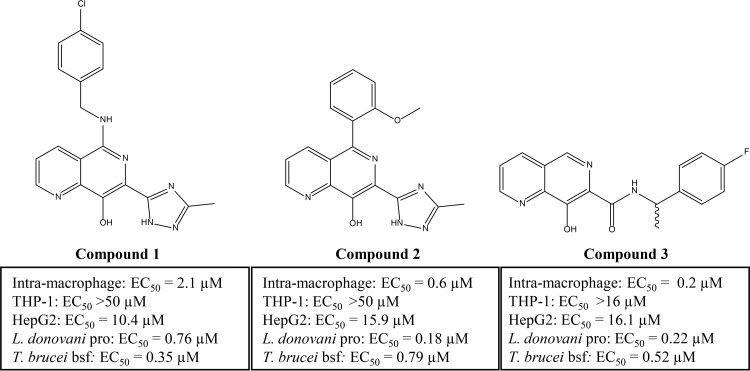
Chemical structures and activities of three 8-HNT compounds. Chemical structures of 8-HNT compound 1 (DDD01007886) (TCMDC-143180; GSK1363355A), compound 2 (DDD01011700) (GSK3454797A), and compound 3 (DDD01012232) (GSK3454397A) are shown. Potencies of compounds against L. donovani promastigotes (pro), L. donovani intramacrophage amastigotes, T. brucei bloodstream forms (bsf), THP-1 cells, and HepG2 cells are shown; data are from ≥3 independent replicates.

Screening of GSK's 1.8-million-compound library against L. donovani axenic amastigotes, followed by secondary screening of “hits” in a L. donovani intramacrophage assay, identified a total of 351 active compounds comprised of 33 compound series and 75 singletons (10). One of these active compounds, an 8-hydroxy naphthyridine (TCMDC-143180) (compound 1) (Fig. 1), demonstrated reasonable potency against both the mammalian (intracellular amastigotes) (50% effective concentration [EC_50_], 2.1 μM) and insect (promastigote) (EC_50_, 0.76 μM) stages of L. donovani. In addition, compound 1 was also active against T. brucei (bloodstream form) (EC_50_, 0.32 μM) while demonstrating limited activity against mammalian cell lines (THP-1 or HepG2) (Fig. 1).

Based upon these promising findings, and the fact that related 8-hydroxyquinoline derivatives have already been shown to demonstrate some moderate activity against both L. donovani and T. cruzi (11, 12), compound 1 was used as a start point for subsequent medicinal chemistry. The principal aims driving compound development were to improve potency, selectivity against HepG2 cells, and aqueous solubility. As a result, compounds 2 and 3 were developed, with both compounds demonstrating improved activity against L. donovani in an intramacrophage assay although similar levels of activity against T. brucei (Fig. 1). Full details of the medicinal chemistry leading to the development of compounds 2 and 3 will be reported elsewhere.

### Genome-wide RNAi screens reveal genes encoding putative cation transporters.

**FIG 2:**
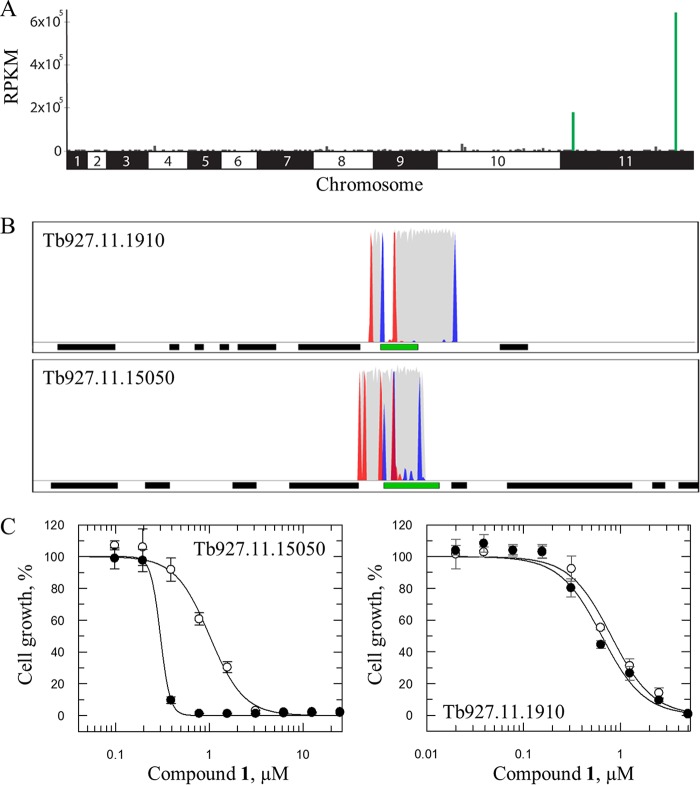
Genome-wide RNAi screening of 8-HNT compounds to identify putative cation transporters in T. brucei. (A) Genome-wide map indicating RIT-seq hits from screening of compound 1. Multiple RIT-seq fragments represent primary hits, indicated in green. Other loci with mapped reads are indicated in gray. RPKM, reads per kilobase of transcript per million mapped reads. (B) Hits on individual genes (indicated in green). Other protein-coding sequences are indicated as black bars. Red peaks, forward reads with RNAi construct barcodes; blue peaks, reverse reads with RNAi construct barcodes; gray peaks, all other reads. (C) Dose-response curves of compound 1 against uninduced (●) and induced (○) Tb927.11.15050 and Tb927.11.1910 knockdown bloodstream trypanosomes. Results are the means ± standard deviations of data from three independent experiments.

An understanding of the MoA or molecular targets of compounds can facilitate the development of more potent and selective compounds. Our studies focused on determining the MoA of 8-HNT compounds in T. brucei and L. donovani. Genome-wide RNA interference (RNAi) screens have proven highly effective in determining drug MoA (13–15). Although these screens do not identify classical drug targets, this methodology can identify drug resistance determinants in T. brucei that assist in the determination of drug MoA. With this in mind, typically lethal concentrations of compounds 1 to 3 (equivalent to 2× to 3× the respective EC_50_s) were used to screen a genome-scale T. brucei bloodstream-form RNAi library, and drug-resistant populations were then subjected to RNAi target sequencing (RIT-seq) (16). During screening under tetracycline induction, each trypanosome produces double-stranded RNA (dsRNA) from an integrated RNAi target fragment, and the resulting target knockdown has the potential to confer a growth advantage under drug selection. RIT-seq then generates a readout that identifies the RNAi target fragments responsible. High-throughput sequencing of the selected populations following screens with compounds 1 to 3 provided between 2.1 million and 3.4 million paired-end reads, with 96 to 98% of reads mapping to the reference genome (Fig. 2A; see also Tables S1 to S3 in the supplemental material). A putative cation transporter (Tb927.11.15050) was identified as a prominent “hit” in all three screens, while screening with compounds 1 and 2 identified a second putative cation transporter (Tb927.11.1910) (Fig. 2B). Additional hits, principally from the screen with compound 1, were identified, including glycosomal ABC transporter 3 (GAT3) (Tb927.11.1070) (17) and subunits of the vacuolar-type H^+^-ATPase (V-ATPase) (18) (summarized in Tables S1 to S3).

To validate the role of the putative cation transporters, independent, inducible, hairpin RNAi knockdown strains were generated for each transporter. Quantitative real-time PCR (qRT-PCR) confirmed that the transcript levels for both genes were reduced by between 50 and 70% without growth defects (see Fig. S1 in the supplemental material). In addition, both native genes were tagged with C-terminal 12×Myc tags in the RNAi strains, and knockdown of the tagged proteins was demonstrated by protein blotting (Fig. S1). Knockdown of Tb927.11.15050 led to a >3-fold increase in the EC_50_ for compound 1, while knockdown of Tb927.11.1910 resulted in no marked change in sensitivity (EC_50_ of 0.6 ± 0.05 μM compared to 0.85 ± 0.05 μM for wild-type [WT] trypanosomes) (Fig. 2C).

### Tb927.11.15050 and Tb927.11.1910 encode Golgi-localized putative zinc transporters.

**FIG 3:**
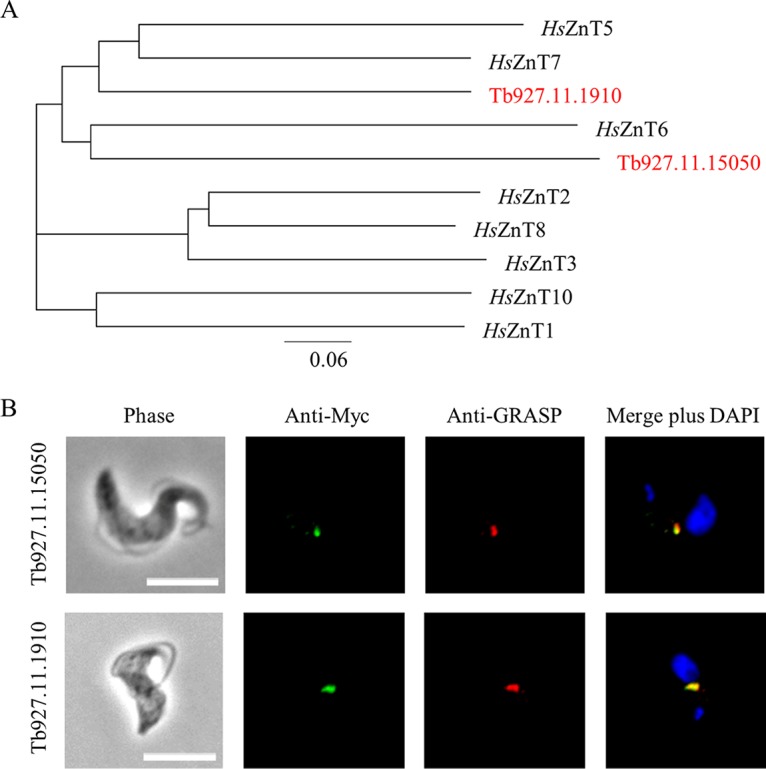
Putative cation transporters share homology with mammalian ZnT transporters and are localized to the Golgi apparatus. (A) Phylogenetic analysis of the proteins encoded by Tb927.11.15050 and Tb927.11.1910 and their relationship to members of the human ZnT zinc transporter family. (B) Myc tags (12×) were introduced to the C termini of each putative cation transporter (Tb927.11.15050 and Tb927.11.1910). Tagged versions of these putative transporters (green) were found to colocalize with GRASP (red), a known marker of the Golgi apparatus of bloodstream trypanosomes. Cells were also stained with 4′,6-diamidino-2-phenylindole (DAPI). Bars, 5 μm.

To gain insight into the function(s) of the two putative cation transporters, phylogenetic and domain analyses of the proteins encoded by Tb927.11.15050 and Tb927.11.1910 were undertaken. Amino acid sequence analysis predicted that both proteins maintained 6 membrane-spanning domains (see Fig. S1 in the supplemental material) and that both proteins were also closely related to a number of zinc transporters, including the well-characterized human ZnT (zinc transporter) family (19). The principal function of the ZnT family is to transport zinc out of the cytoplasm, in contrast to the ZIP (ZRT, IRT-like protein) family of transporters that are responsible for zinc uptake into the cytoplasm (19). The Tb927.11.15050-encoded protein was most closely related to ZnT6, while the Tb927.11.1910-encoded protein was most closely related to ZnT5 and ZnT7 (Fig. 3A). In human cells, ZnT5 to -7 are localized to the Golgi apparatus and involved in the transport of zinc into the organelle (20, 21). Indeed, both the Tb927.11.15050 and Tb927.11.1910 proteins fused with a C-Myc tag colocalized with the Golgi marker GRASP (Golgi reassembly stacking protein) (22) in bloodstream trypanosomes (Fig. 3B). Collectively, these data suggest that the proteins encoded by Tb927.11.15050 and Tb927.11.1910 likely function as zinc transporters facilitating the uptake of zinc into the Golgi apparatus.

### Zn^2+^ or Fe^2+^ reduces the potency of 8-HNT compounds against T. brucei and L. donovani.

**FIG 4:**
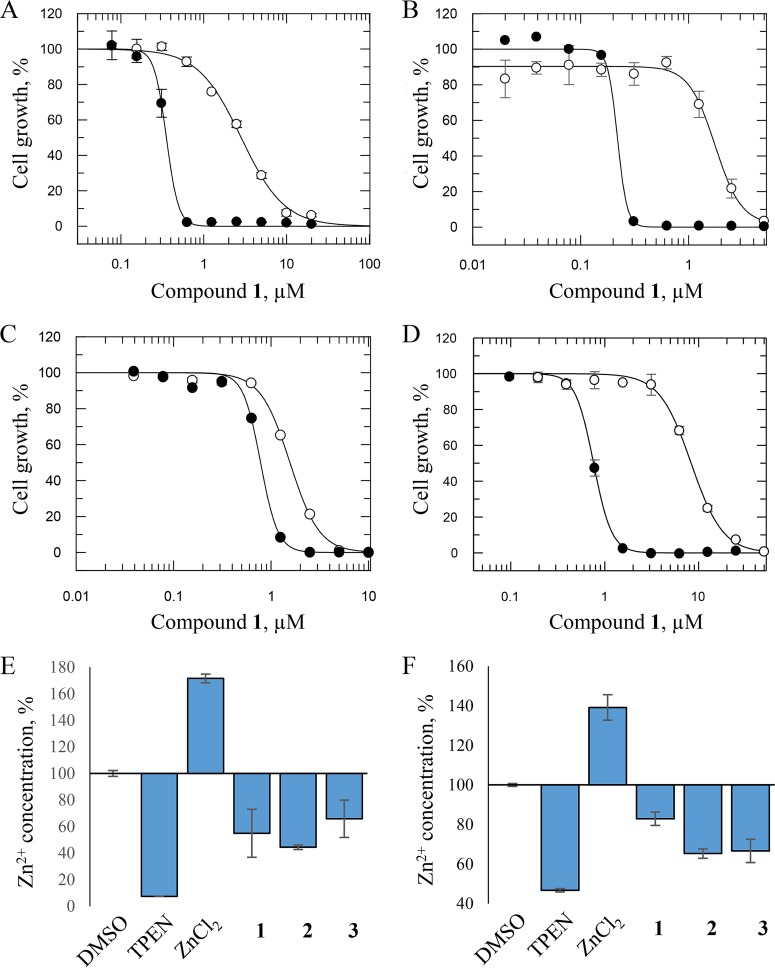
ZnCl_2_ or FeCl_2_ protects parasites, and intracellular Zn^2+^ levels are decreased in the presence of 8-HNT compounds. (A and B) Dose-response curves of compound 1 in the presence (○) and absence (●) of ZnCl_2_ (A) or FeCl_2_ (B) in T. brucei bloodstream-form parasites. In the presence of ZnCl_2_ (200 μM), EC_50_s shifted from 0.35 ± 0.01 μM to 2.8 ± 0.12 μM. In the presence of FeCl_2_ (10 μM), EC_50_s shifted from 0.22 ± 0.01 μM to 1.8 ± 0.18 μM. (C and D) Dose-response curves of compound 1 in the presence (○) and absence (●) of ZnCl_2_ (C) or FeCl_2_ (D) in L. donovani promastigotes. In the presence of ZnCl_2_ (100 μM), EC_50_s shifted from 0.76 ± 0.008 μM to 8.3 ± 0.32 μM. In the presence of FeCl_2_ (100 μM), EC_50_s shifted from 0.78 ± 0.03 μM to 1.6 ± 0.05 μM. All EC_50_s represent the means ± standard deviations from three independent experiments. (E and F) Intracellular measurement of zinc as a percentage in T. brucei bloodstream parasites normalized to the dimethyl sulfoxide (DMSO) control (100%) (E) and intracellular measurement of zinc as a percentage in L. donovani promastigotes normalized to the DMSO control (100%) (F). Levels of intracellular zinc were measured by using the fluorescent zinc reporter FluoZin-3 (final concentration, 5 μM), as described in Materials and Methods, in the presence of the zinc chelator TPEN (8 μM), exogenous ZnCl_2_ (100 μM), or compounds 1 to 3 (5 μM). All values represent the means ± standard errors of the means for at least 3 experiments.

The data described above suggested that the depletion of zinc import to the Golgi apparatus reduced the efficacy of the 8-HNT compounds against T. brucei. Therefore, we hypothesized that 8-HNT compounds were reducing zinc levels in trypanosomes; zinc transporter knockdown could reduce potency by increasing cytoplasmic zinc concentrations. To test this hypothesis, we added exogenous zinc to parasites and asked whether this reduced the potency of the 8-HNT compounds. In the first instance, we established the level of ZnCl_2_ that could be added to T. brucei without impacting cell growth and viability (200 μM ZnCl_2_) (see Fig. S2 in the supplemental material). Next, the EC_50_ for compound 1 was determined in the presence and absence of 200 μM ZnCl_2_. The addition of exogenous Zn^2+^ led to an 8-fold decrease in the potency of compound 1, with an EC_50_ of 2.8 ± 0.1 μM, compared to 0.36 ± 0.09 μM in the absence of Zn^2+^ (Fig. 4A). Similar zinc-dependent shifts in potency were observed for all three 8-HNT compounds (Table 1). Notably, the Zn^2+^-dependent shifts in potency observed with 8-HNT compounds in T. brucei mimic the modulation of potency of a known zinc chelator [*N*,*N*,*N*′,*N*′-tetrakis(2-pyridylmethyl)ethane-1,2-diamine (TPEN)] (Fig. S2). Our culture medium is supplemented with 10% fetal bovine serum (FBS), and it is well established that serum contains many proteins that bind zinc and other metals (23). Therefore, it is likely that significant amounts of Zn^2+^ are required to saturate the metal binding capacity of these serum proteins prior to impacting the potency of 8-HNT compounds.

**TABLE 1:** EC_50_s for 8-HNT compounds against T. brucei in the presence and absence of divalent transition metals^*a*^

Divalent cation	Compound 1			Compound 2			Compound 3		
	Mean EC_50_ (μM) ± SD		Shift (fold)	Mean EC_50_ (μM) ± SD		Shift (fold)	Mean EC_50_ (μM) ± SD		Shift (fold)
	WT	Plus cation		WT	Plus cation		WT	Plus cation	
CaCl_2_	0.3 ± 0.8	0.4 ± 0.1	1.1	1.2 ± 0.04	1.3 ± 0.06	1.0	0.8 ± 0.1	0.7 ± 0.04	0.9
CuCl_2_	0.3 ± 0.08	0.4 ± 0.005	1.1	1.4 ± 0.07	1.3 ± 0.06	0.9	0.8 ± 0.05	0.8 ± 0.06	1.1
FeCl_2_	0.2 ± 0.01	1.8 ± 0.2	8.0	0.7 ± 0.06	1.6 ± 0.05	2.4	0.9 ± 0.03	3.5 ± 0.3	3.7
MgCl_2_	0.4 ± 0.01	0.4 ± 0.03	1.1	0.8 ± 0.05	0.9 ± 0.02	1.1	0.8 ± 0.05	0.7 ± 0.05	0.9
MnCl_2_	0.4 ± 0.01	0.4 ± 0.02	1.0	1.4 ± 0.09	1.2 ± 0.05	0.8	0.8 ± 0.05	1.01 ± 0.04	1.3
ZnCl_2_	0.4 ± 0.01	2.8 ± 0.1	8.0	0.8 ± 0.05	15.6 ± 0.7	19.7	0.5 ± 0.03	6.3 ± 0.2	12.0

a EC_50_s represent the means ± standard deviations for at least 3 experiments.

The effect of other divalent cations (Ca^2+^, Cu^2+^, Fe^2+^, Mg^2+^, and Mn^2+^) on the potency of the 8-HNT analogues was also assessed in T. brucei (Table 1). The addition of 10 μM exogenous FeCl_2_ to culture medium, the highest concentration of Fe^2+^ tolerated by bloodstream T. brucei parasites, led to considerable increases in the EC_50_s of the three compounds. The most marked shift in potency was seen with compound 1, where the EC_50_ was increased from 0.22 ± 0.01 μM to 1.8 ± 0.2 μM (8-fold) in the presence of FeCl_2_ (Fig. 4B). In contrast, the addition of tolerated levels of all other divalent cations had little or no impact on the potency of 8-HNT compounds. As a control, and as expected, the addition of exogenous divalent cations, including Fe^2+^ and Zn^2+^, failed to alter the potency of a specific ornithine decarboxylase inhibitor, difluoromethylornithine (DFMO), against T. brucei (Fig. S2).

8-HNT compounds demonstrated promising activity against both intramacrophage (amastigote) and insect-stage (promastigote) L. donovani parasites. To determine if the addition of exogenous zinc also shifted the potency of these compounds for L. donovani, EC_50_s for all three 8-HNT analogues against promastigotes were determined in the presence and absence of ZnCl_2_. The addition of 100 μM Zn^2+^ led to an 11-fold reduction in the potency of compound 1 (Fig. 4C) and even larger shifts in the potencies of compounds 2 and 3 (Table S4). Similarly, the addition of 100 μM FeCl_2_ reduced the potency of 8-HNT compounds for L. donovani (Fig. 4D and Table S4). These data suggest that 8-HNT compounds are likely to share similar MoA in both T. brucei and L. donovani.

### 8-HNT compounds deplete levels of intracellular Zn^2+^.

To further explore our hypothesis that 8-HNT compounds reduce Zn^2+^ levels in parasites, the cell-permeable fluorescent reporter reagent FluoZin-3 was used to monitor intracellular levels of Zn^2+^ in the presence or absence of all three compounds. The fluorescence-based assay was responsive to gross changes in intracellular levels of zinc in T. brucei bloodstream forms or L. donovani promastigotes, as demonstrated by the addition of exogenous ZnCl_2_ (100 μM) or by the addition of the zinc chelator TPEN (8 μM) (Fig. 4E). Like TPEN, the addition of the 8-HNT compounds (5 μM) resulted in a decrease in fluorescence, indicating a depletion of the intracellular levels of zinc in both parasites (Fig. 4E and F). It should be noted that our EC_50_s determining parasite viability are measured over a 72-h period, while our intracellular zinc assays are carried out within a 30-min period. Therefore, it is not possible to directly correlate the amounts of Zn depleted in these assays with those in our viability assays.

Initial screening demonstrated that 8-HNT compounds are moderately toxic to human THP-1 monocytes and HepG2 cells, providing a potentially tolerable therapeutic window (Fig. 1). Our data thus far suggested that 8-HNT compounds impacted Zn^2+^ and Fe^2+^ levels in multiple protozoan parasites. Therefore, we investigated the possibility that 8-HNT compounds could be similarly affecting mammalian cells. First, the addition of 100 μM ZnCl_2_ shifted the EC_50_ of compound 2 for HepG2 cells 7-fold, from 47.8 ± 3.6 μM to 318.9 ± 22 μM (see Fig. S3 in the supplemental material). Second, the FluoZin-3-based assay showed that all three 8-HNT compounds deplete intracellular levels of Zn^2+^ in HepG2 cells (Fig. S3). These data strongly suggest that 8-HNT compounds have a MoA involving the depletion of divalent cations within protozoan and mammalian cells and potentially other cell types.

To determine whether 8-HNT compounds act by entering cells and directly depleting intracellular Zn^2+^ or by sequestering extracellular Zn^2+^ in media, the uptake of compound 1 by L. donovani promastigotes was measured. Using ultraperformance liquid chromatography-tandem mass spectrometry (UPLC-MS/MS), the uptake of compound 1 (5 μM) by mid-log-phase parasites was determined following 30-min incubations at 37°C and 4°C. At 37°C, compound 1 was found to accumulate within cells to levels 296 ± 69 times higher than those seen in the supernatant (equivalent *Kp* [permeability coefficient] values). In contrast, at 4°C, the accumulation of compound 1 in promastigotes was significantly reduced, to levels 29 ± 13 times higher than those in supernatants. Collectively, these data suggest that 8-HNT compounds are likely to be transported rather than to diffuse into cells and mediate their effects on intracellular levels of Zn^2+^ directly from within cells.

### 8-HNT compounds are divalent cation chelators.

**FIG 5:**
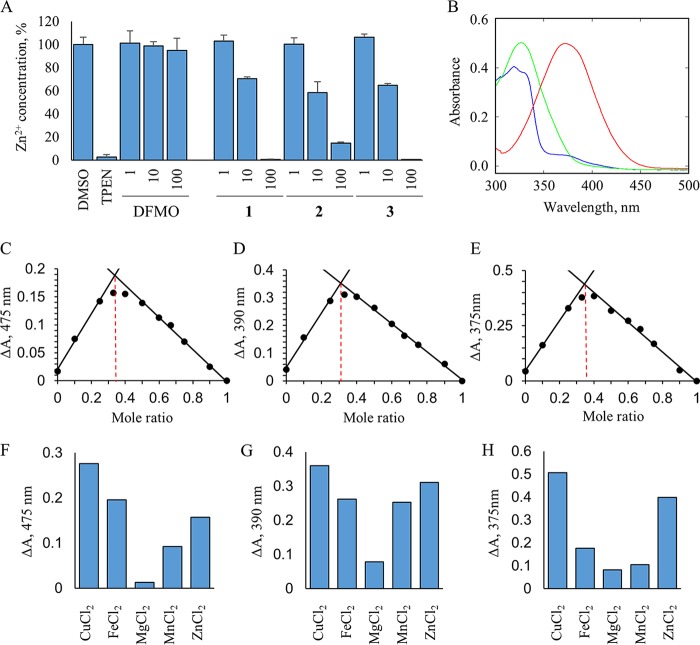
Characterization of Zn^2+^ binding to 8-HNT compounds in a cell-free system. (A) Levels of zinc were measured by using the fluorescent zinc reporter FluoZin-3 salt (final concentration, 5 μM), as described in Materials and Methods, in the presence of the zinc chelator TPEN (8 μM), exogenous ZnCl_2_ (100 μM), DFMO (1, 10, and 100 μM), or compounds 1 to 3 (1, 10, and 100 μM). Data were normalized to Zn^2+^ levels in the presence of a DMSO control (100%). All values represent the means ± standard errors of the means for 3 independent experiments. (B) UV-Vis spectra of 50 μM compound 1 (red), compound 2 (green), and compound 3 (blue). (C to E) Job's plots of Δ absorbance at 475 nm for compound 1, 390 nm for compound 2, and 375 nm for compound 3 relative to fixed molar ratios of compound to Zn^2+^. The intercepts at molar fractions of 0.34 for compound 1, 0.31 for compound 2, and 0.35 for compound 3, illustrated by dashed red lines, are indicative of a 2:1 stoichiometry between 8-HNT compounds and Zn^2+^, respectively. (F to H) The Δ absorbance values at fixed molar ratios of compound to cation of 0.33 were plotted for each 8-HNT compound and a range of divalent cations (Cu^2+^, Fe^2+^, Mg^2+^, Mn^2+^, and Zn^2+^).

The potential for 8-HNT compounds to bind directly to Zn^2+^ in a cell-free environment was assessed. The tetrapotassium salt of the FluoZin-3 reporter reagent was incubated in the presence of ZnCl_2_ (100 μM) and either TPEN (10 μM) or compounds 1 to 3 (1 to 100 μM) (Fig. 5A). As expected, the addition of TPEN resulted in a reduction in the level of fluorescence, consistent with direct competition with the FluoZin-3 probe for Zn^2+^ binding by this established zinc chelator. The addition of 8-HNT compounds 1 to 3 to the assay mixture also resulted in a dose-dependent reduction in the level of fluorescence, again signifying direct competition with the probe for Zn^2+^ binding (Fig. 5A). In contrast, the control compound (DFMO) did not affect the fluorescence levels, suggesting that it does not bind to Zn^2+^.

The data described above strongly suggest that 8-HNT compounds act principally as chelators of Zn^2+^ and Fe^2+^. Thus, the formation of metal ion–8-HNT complexes was investigated by using spectrophotometric wavelength scanning. Each 8-HNT compound has a characteristic spectrum in the absence of divalent cations (Fig. 5B). The addition of increasing concentrations of specific divalent cations alters this spectrum, which is indicative of the formation of new metal ion–8-HNT complexes. Spectral changes induced by the addition of increasing concentrations of zinc were characterized and quantified for compound 1 (see Fig. S4 in the supplemental material). The addition of Zn^2+^ resulted in an increase in the absorbance at 475 nm (Fig. S4), confirming that, as suspected, compound 1 forms a complex with Zn^2+^. Plotting the change in the absorbances at 475 nm for a fixed concentration of compound 1 (50 μM) and increasing concentrations of Zn^2+^ demonstrated that binding of Zn^2+^ is saturable at 25 μM (Fig. S4), indicating a 2:1 complex of compound 1-Zn^2+^.

The stoichiometry of the complexes formed between the three 8-HNT compounds and Zn^2+^ was further investigated by measuring the absorbance at specific wavelengths while changing the molar ratio of each 8-HNT compound to Zn^2+^ (Fig. 5C to E). This analysis is commonly referred to as Job's plot (24). Changes of the absorption at 475, 390, and 375 nm are indicative of cation complex formation with compounds 1 to 3, respectively. The specific absorbance at these wavelengths was determined and plotted against the molar ratio of the compound/cation to determine complex stoichiometry. Peaks of absorbance at these wavelengths were achieved with molar ratios of compounds 1 to 3 to Zn^2+^ of 0.34, 0.31, and 0.35, respectively. Since a molar ratio of 0.33 indicates the formation of a 2:1 stoichiometric complex, the data are consistent with all three 8-HNT compounds forming a 2:1 complex with Zn^2+^. Similarly, this 2:1 stoichiometry was observed for all complexes formed between the 8-HNT analogues and the other divalent cations tested (Fig. 5).

It is not possible to accurately calculate the binding affinity (*K_d_* [dissociation constant]) for components of 2:1 complexes; however, Job's plots can provide some indication of the comparative strengths of binding between 8-HNT compounds and different divalent cations (24). Here, the absorbance at a fixed molar ratio of 0.33 (compound/cation) was measured and compared for each 8-HNT compound-cation complex (Fig. 5F to H). This analysis revealed subtle differences in the binding preferences of 8-HNT analogues for specific divalent cations. However, all three 8-HNT compounds formed complexes most readily with Cu^2+^ and least effectively with Mg^2+^. In keeping with our above-described data, all three analogues also bound strongly to Zn^2+^ and Fe^2+^. Based on the *in vitro* data described above, the strength of 8-HNT binding to Cu^2+^ was surprising. An explanation for why the addition of Cu^2+^ may have failed to shift the potency of 8-HNT against T. brucei *in vitro* is presented below. In conclusion, the cytotoxic effects of 8-HNT compounds are due to the chelation of divalent cations.

## DISCUSSION

Several lines of evidence presented here establish that 8-HNT compounds chelate divalent cations, principally Cu^2+^, Zn^2+^, and Fe^2+^, and that this chelation forms the primary basis of their cytotoxicity in trypanosomatid parasites and mammalian cells. First, RNAi knockdown of two putative cation transporters, closely related to human ZnT transporters involved in the uptake of zinc into the Golgi apparatus, are associated with resistance to 8-HNT analogues. Second, the addition of exogenous Zn^2+^ and Fe^2+^ reduces the potency of 8-HNT compounds for L. donovani, T. brucei, and HepG2 cells. Third, 8-HNT compounds deplete intracellular levels of Zn^2+^ in L. donovani and HepG2 cells. Finally, spectrophotometric wavelength scanning demonstrates that 8-HNT compounds bind directly to Cu^2+^, Zn^2+^, and Fe^2+^ to form a 2:1 stoichiometric complex with these divalent cations.

The putative cation transporters linked to decreased susceptibility to 8-HNT compounds in the RNAi screens are closely related to the mammalian ZnT family of vesicular zinc transporters, which are primarily associated with Zn^2+^ sequestration into intracellular compartments (25) and, consequently, also act to lower Zn^2+^ concentrations in the cytoplasm (26). The putative transporters encoded by Tb927.11.15050 and Tb927.11.1910 localize specifically to the Golgi apparatus of bloodstream trypanosomes. Recent studies have demonstrated that the Golgi apparatus, alongside the endoplasmic reticulum (ER), acidocalcisomes, and mitochondria, serves as an intracellular zinc store (27, 28). Thus, the knockdown of Golgi-specific zinc importers likely leads to an increase in the concentration of free zinc within the cytoplasm. Since the cell-permeable zinc probe used in our studies merely allowed us to measure significant changes in total intracellular Zn^2+^ levels, it was not possible to directly measure the likely small changes in cytosolic Zn^2+^ that may result from the knockdown of these Golgi-localized Zn^2+^ importers. In the presence of a chelator, decreased export to the Golgi apparatus may act to protect essential zinc-dependent functions within the cytoplasm. Indeed, the transcript levels of ZnT family members 5, 6, and 7, most closely related to the transporters identified in this study, were significantly reduced in THP-1 monocytes treated with the zinc chelator TPEN (29). An alternative mechanism of resistance to chelators such as 8-HNT compounds would be to reduce the levels of plasma membrane-localized ZnT1 homologues, principally responsible for the efflux of zinc out of the cell. No such transporters were identified in our RNAi screens, and subsequent genome analyses failed to identify ZnT1 homologues in T. brucei.

In an attempt to tease out additional mechanisms of resistance to 8-HNT analogues, L. donovani promastigotes were exposed to stepwise increasing concentrations of compound 2 over several months, with the rationale that resistance determinants could be identified by whole-genome sequencing of the resistant population. However, over a 9-month period, we could not develop any resistance to compound 2 in these parasites. Our failure to develop resistance to this 8-HNT compound in L. donovani is particularly noteworthy. In our experience over several years, generation of resistance in L. donovani promastigotes *in vitro* is eminently achievable (30–33), and compound 2 represents our first failure. This observation is entirely consistent with chelation as the MoA of this compound, because the pleiotropic functions of zinc and iron within the cell are likely to leave parasites unable to generate mutations capable of alleviating all the effects of metal extraction from multiple protein targets.

Additional hits from our RNAi library screens included subunits of the V-ATPase. V-ATPases are involved in the generation of a proton gradient required for the ATP-dependent transport of Zn^2+^ and Fe^2+^ into organelles, including the Golgi apparatus (34–37). It is tempting to suggest that this role in the sequestration of divalent cations may explain the relationship between V-ATPase subunit knockdown and 8-HNT resistance.

Our RNAi screens led us to focus on the link between 8-HNT compounds and effects on zinc homeostasis; however, our subsequent studies clearly demonstrate that 8-HNT compounds are also strong chelators of Fe^2+^. Undoubtedly, Leishmania and T. brucei have a considerable requirement for both cations. It is well established that changes in iron availability have a strong influence on the growth and virulence of pathogens, while iron deprivation by various means is an important component of the host's first-line defense against infection (38). The zinc chelator TPEN has been shown to inhibit L. donovani *in vitro* (39, 40). Those studies demonstrated that zinc depletion led to increased levels of reactive oxygen species in TPEN-treated promastigotes. Metal ions such as Zn^2+^ and Fe^2+^ play a key role in redox reactions; as a consequence, chelation of metal ions can lead to elevated levels of oxidative stress. Several iron chelators (41), including deferoxamine (42), have also been shown to inhibit the growth of both L. donovani and T. brucei *in vitro*. Deferoxamine also inhibits the growth of L. major *in vitro* but was never tested *in vivo* due to the accompanying cytotoxicity against mammalian cells (43).

**FIG 6:**
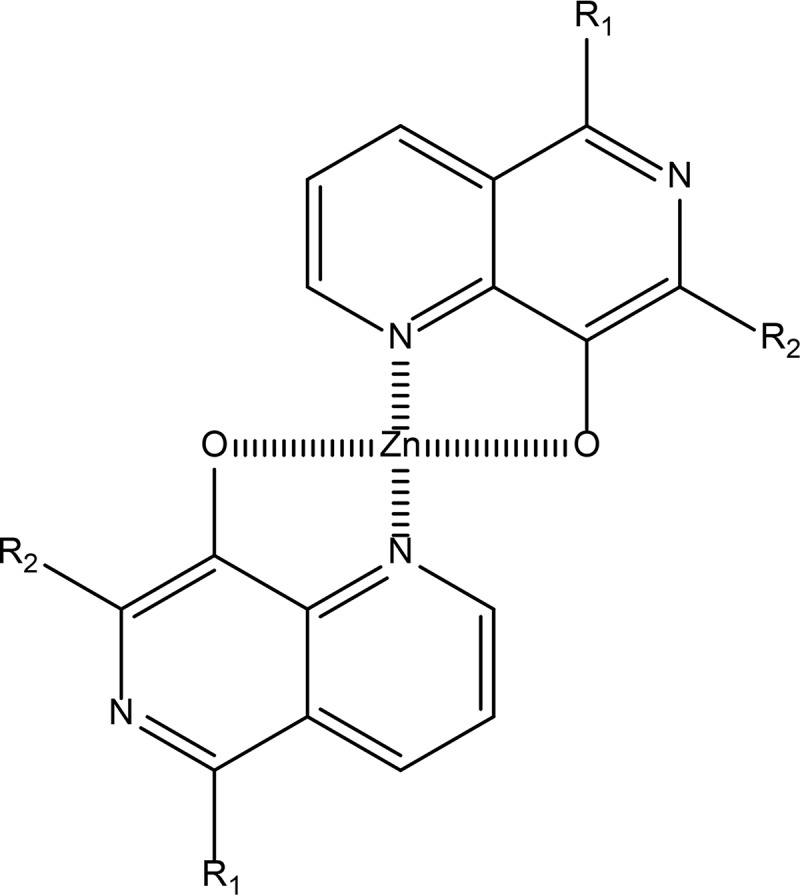
Proposed structure of the 2:1 complex formed between 8-HNT compounds and Zn^2+^.

Our spectrophotometric wavelength scanning studies demonstrate that 8-HNT analogues form 2:1 stoichiometric complexes with divalent cations (Fig. 6). The coordination of metal ions by these compounds is very similar to the complexes formed between 8-hydroxyquinolines and metals, and it is evident that 8-HNT compounds share a structural motif with this compound class (44). Collectively, our studies illustrate that three 8-HNT analogues demonstrate subtle differences in their preferences for binding specific divalent cations. Of particular note, this approach illustrated the preference of 8-HNT compounds for binding Cu^2+^. Copper is known to form highly stable complexes with cysteine (45) and catalyzes the oxidation of cysteine (46). Bloodstream trypanosomes are cultured in HMI9-T medium, which is known to contain high levels of cysteine as well as bathocuproine, a copper chelator (47). It is highly likely that the bolus of Cu^2+^ added to our culture medium is rapidly reduced by reaction with cysteine to Cu^+^ (48) and that the resulting Cu^+^ is then chelated by bathocuproine, preventing Cu^+^ from entering the cell (49). Thus, while our cell-based data place the chelation of Zn^2+^ and Fe^2+^ at the heart of the cytotoxicity of these compounds, we cannot rule out the possibility that chelation of Cu^2+^ may also play a significant role.

The identification of chelation as the main driver for cytotoxicity is likely to present a significant challenge for the chemotherapeutic development of this chemical series, specifically maintaining the potency of 8-HNT compounds while ensuring a sufficient therapeutic window. As it stands, our best 8-HNT compounds maintain an ∼100-fold therapeutic window; clearly, work will be required to improve selectivity. In the search for strategies to treat metal overload diseases, there is growing interest in the development of “prochelators” (50–52), molecules that have little affinity for metal ions until they undergo activation in response to specific stimuli. Activation generally involves the removal of a protective moiety by stimuli such as enzyme activation, oxidative stress, or reducing environments. Our present study joins several others in illustrating the vulnerability of kinetoplastid parasites to metal ion depletion. In the future, it may be possible to employ a prochelation strategy to develop potent and safe 8-HNT analogues that are activated at the site of infection.

## MATERIALS AND METHODS

### 8-HNT compounds.

All 8-HNT test compounds were synthesized by medicinal chemists within the GlaxoSmithKline and University of Dundee collaboration funded by the Wellcome Trust. Details of chemical syntheses for compound 1 (TCMDC-143180), compound 2, and compound 3 will be provided in a subsequent publication.

### Compounds.

All compounds were of the highest quality and are as follows: TPEN [*N*,*N*,*N*′,*N*′-tetrakis(2-pyridylmethyl)ethylenediamine; Sigma], FluoZin-3 AM (ThermoFisher), FluoZin-3 tetrapotassium salt (ThermoFisher), pluronic F-127 (ThermoFisher), Fluo-4 AM (ThermoFisher), probenecid (ThermoFisher), and ionomycin (Sigma).

### Cell lines and culture conditions.

The clonal Leishmania donovani cell line LdBOB (derived from MHOM/SD/62/1S-CL2D) was grown as promastigotes at 28°C, as described previously (53). Bloodstream-form T. brucei MiTat 1.2 clone 221a and its derived subline, 2T1, were cultured at 37°C in the presence of 5% CO_2_ in HMI9-T medium, as described previously (54). The human carcinoma HepG2 cell line was grown at 37°C with 5% CO_2_ in minimal Eagle's medium (MEM) with GlutaMAX (1×; Sigma-Aldrich), nonessential amino acids (Sigma-Aldrich), and 10% FBS. Human HepG2 and THP-1 cells used in this study were ethically sourced from the ATCC.

### *In vitro* drug sensitivity assays.

L. donovani intramacrophage (7), HepG2 (55), T. brucei (56), and L. donovani promastigote (57) compound sensitivity assays were carried out as described previously. Human HepG2 and THP-1 cells used in these assays were provided by the ATCC. Data were processed by using GRAFIT (version 5.0.4; Erithacus Software) and fitted to a 2-parameter equation to obtain the effective concentration inhibiting growth by 50% (EC_50_). Data are presented as the means of results from at least two independent experiments.

### RIT-seq screening, Illumina sequencing, and analysis.

The RNAi library screens were performed as described previously (16). Tetracycline induction (24 h) of genome-wide RNAi initiates the formation of gene-specific dsRNAs, leading to target knockdown. The population is then screened with compounds to identify parasites where specific RNAi target knockdown has conferred compound resistance. Sequencing (RIT-seq) is then used to generate a genome-wide readout from the compound-resistant parasite population. Libraries were maintained under blasticidin (1 μg ml^−1^) and phleomycin (1 μg ml^−1^) selection in 150 ml medium with a minimum of 2 × 10^7^ cells. Following tetracycline (1 μg ml^−1^) induction for 24 h, compound 1 (448 nM), compound 2 (1.8 μM), and compound 3 (1.2 μM) were added to cultures at concentrations equivalent to 2× to 3× their respective EC_50_s. Cultures were supplemented with fresh compound and tetracycline as required. Based on cell growth, concentrations of compounds 2 and 3 were increased during library selection to final concentrations of 5 μM and 3.6 μM, respectively. This increase in the compound concentration was required to ensure that the surviving parasite population was truly resistant and not merely tolerating the presence of the compound(s). This step ensures that “hits” identified in subsequent analyses are specifically related to resistance mechanisms. DNA was extracted from compound-selected cells on days 8, 13, and 20 for compounds 1, 3, and 2, respectively.

RNAi target fragments were amplified from compound-selected parasites by PCR using the Lib2f and Lib2r primers (16). For high-throughput identification of fragments, the PCR products were fragmented and sequenced by using an Illumina HiSeq platform at BGI (Beijing Genomics Institute). Reads were mapped to the T. brucei 927 reference genome (v9.0; TriTrypDB [http://tritrypdb.org/]) with Bowtie 2 software (58), using the following parameter: very-sensitive-local-phred33. The subsequent alignment files were manipulated with SAMtools (59) and a custom script to identify reads with barcodes (GCCTCGCGA) (16). Total and barcoded reads were then quantified by using the Artemis genome browser (60).

### Phylogenetic analysis.

Phylogenetic analysis of the proteins encoded by Tb927.11.1910 and Tb927.11.15050 was performed by using NCBI protein blast. Sequences for the human ZnT proteins were downloaded from the NCBI database and aligned by using ClustalX. A protein feature visualization tool, Protter, was used to identify and display transmembrane domains (61).

### Stem-loop RNAi and tagging vector construction.

Stem-loop RNAi constructs were assembled as described previously (54, 62). PCR primers were designed by using RNAit software (63). For HL2 (Tb927.11.15050), primers HL2FW and HL2RV were used to generate a 533-bp gene fragment, and for HL3 (Tb927.11.1910), primers HL3FW and HL3RV were used to generate a 513-bp gene fragment. The accuracy of all assembled constructs was verified by sequencing. Assembled constructs were linearized by using AscI prior to transfection into bloodstream T. brucei 2T1 parasites (62). In addition to phleomycin (1 μg ml^−1^), trypanosomes maintaining successfully integrated vectors were selected using hygromycin (2.5 μg ml^−1^) and confirmed to be puromycin (2 μg ml^−1^) resistant. To generate C-terminal 12×Myc tagging vectors for Tb927.11.15050 (Myc1) and Tb927.11.1910 (Myc2), primers Myc1FW and Myc1RV and primers Myc2FW and Myc2RV were used to generate 924-bp and 1,095-bp fragments, respectively. Fragments were inserted into the pNATx^12xMyc^ construct (54) via HindIII and XbaI restriction sites. The accuracy of the assembled constructs was verified by sequencing, and the constructs were linearized with the restriction endonuclease MfeI prior to transfection into the above-described HL2 and HL3 trypanosomes. Trypanosomes maintaining successfully integrated vectors were selected with blasticidin (1 μg ml^−1^).

### qRT-PCR.

RNA was isolated from T. brucei bloodstream-form parasites by using an RNeasy purification kit (Qiagen), and cDNA was synthesized by using a high-capacity RNA-to-cDNA kit (Applied Biosystems). qRT-PCR mixtures consisted of 2 μl (80 ng) cDNA, 10 μl Brilliant III Ultra-Fast QPCR master mix (Agilent Technologies), 1 μl (500 nM) of the forward and reverse primers, 0.3 μl (30 nM) of reference dye, and nuclease-free water. Analysis was conducted by using an Agilent Mx3005P machine under the following cycling conditions: 95°C for 3 min, 40 cycles of 95°C for 20 s, and then 60°C for 20 s. A reference gene, *TERT* (telomerase reverse transcriptase) (Tb927.11.10190), was used to provide a baseline of transcription levels to allow normalization of the data (64). Relative quantification for the tetracycline-induced RNAi knockdown cell lines was normalized to the value for the corresponding uninduced cell line by using the ΔΔ*C_T_* method. Two experimental clones were used, and Student's unpaired *t* test was used to show significance.

### Western blotting.

T. brucei whole-cell extracts (1 × 10^6^ parasites per lane) were prepared in sample buffer (62 mM Tris [pH 6.8], 10% glycerol, 2.3% SDS, 5% 2-mercaptoethanol) in the absence of boiling, separated by SDS-PAGE, and transferred onto nitrocellulose. After blocking with 5% skimmed milk in phosphate-buffered saline (PBS) for 1 h, blots were incubated for 1 h in the presence of a mouse monoclonal anti-Myc antibody (1:500; Source BioScience), and membranes were washed in PBS containing 0.1% (vol/vol) Tween 20 and then incubated with a secondary antibody (rabbit anti-mouse IgG) (1:10,000; Dako, UK). Immunoblots were developed by using the ECL (enhanced chemiluminescence) plus system from GE Healthcare (Piscataway, NJ, USA).

### Effect of divalent metals on the potency of 8-HNT compounds against T. brucei, L. donovani, and HepG2 cells.

In the first instance, the maximum tolerated concentrations of ZnCl_2_, CaCl_2_, CuCl_2_, MgCl_2_, MnCl_2_, and FeCl_2_ were determined for each cell type. For T. brucei, the maximum concentrations of metal salt having a minimal impact on cell growth were as follows: 200 μM ZnCl_2_, 3 mM CaCl_2_, 5 μM CuCl_2_, 1 mM MgCl_2_, 10 μM MnCl_2_, and 10 μM FeCl_2_. For L. donovani, promastigotes were able to tolerate the addition of 100 μM exogenous FeCl_2_ and ZnCl_2_. HepG2 cells tolerated the addition of 100 μM exogenous ZnCl_2_. Standard drug sensitivity assays were carried out (as described above) for each cell type with compounds 1, 2, and 3 in the presence and absence of the maximum tolerated concentration of metal salt.

### Immunofluorescence.

Immunofluorescence microscopy was carried out according to standard protocols. Briefly, 1 × 10^6^ cells were fixed in 1% formaldehyde in PBS and allowed to dry on coverslips. Cells were permeabilized with 0.5% Triton X-100 in PBS, blocked with 50% FBS in PBS, and incubated with a monoclonal mouse anti-Myc antibody (1:500; Source BioScience) and a polyclonal rabbit anti-GRASP antibody (1:750; a kind gift from Graham Warren). Secondary anti-mouse Alexa Fluor 488 and anti-rabbit Alexa Fluor 468 antibodies (Life Technologies) were used at 1:2,000 dilutions. Cells were mounted in Vectashield (Vector Laboratories) containing the DNA counterstain DAPI (4′,6-diamidino-2-phenylindole). Images were captured by using a Zeiss Axiovert 200M fluorescence microscope and processed with ZenPro (Zeiss).

### Generation of an 8-HNT-resistant L. donovani cell line.

Resistant lines were generated as described previously (31), using compound 2 in L. donovani promastigotes. Starting at a sublethal concentration of 1 μM compound 2 in 3 independent cultures, concentrations were increased in a stepwise manner. However, after a total of 270 days in culture, promastigotes were unable to grow in ≥10 μM compound 2.

### Measurement of intracellular Zn^2+^ levels in 8-HNT-treated L. donovani promastigotes and T. brucei bloodstream forms.

L. donovani promastigotes were grown to 2 × 10^7^ cells ml^−1^ and harvested by centrifugation (10 min at 1,600 × *g*). T. brucei bloodstream forms were grown to 2 × 10^6^ cells ml^−1^ and harvested by centrifugation (5 min at 800 × *g*). Parasites were washed with PBS and resuspended to a final concentration of 2.5 × 10^7^ parasites ml^−1^ in fresh PBS. Cells were incubated with FluoZin-3 (final concentration, 5 μM), pluronic acid (final concentration, 5 μM), and 14 mM glucose (T. brucei only). Parasites were incubated for 60 min at room temperature (RT), washed three times with PBS, and then resuspended in PBS (with 14 mM glucose for T. brucei only) before being aliquoted into a black, clear-bottomed 96-well plate (final concentration of 2.5 × 10^7^ cells ml^−1^). 8-HNT or control compounds were added directly to cells, and fluorescence was measured immediately by using a Pherastar fluorescence spectrophotometer (excitation at 485 nm and emission at 520 nm). Calculations were performed in Excel. Preliminary analysis confirmed that 8-HNT compounds did not quench fluorescence in the absence of FluoZin-3. Parasites were confirmed to be viable by microscopy after the completion of the experiment.

### Measurement of intracellular Zn^2+^ levels in 8-HNT-treated human HepG2 cells.

Prior to experiments, HepG2 cells (1 × 10^4^ cells per well) were plated into a 96-well plate and left overnight to adhere. The following day, cells were washed twice with PBS and then incubated in 200 μl PBS containing FluoZin-3 (final concentration, 5 μM) and pluronic acid (final concentration, 5 μM) for 60 min at RT. Cells were then washed three times with PBS prior to resuspension in 200 μl PBS. Finally, cells were treated with 100 μM 8-HNT compounds or 8 μM TPEN, and fluorescence was measured immediately at an excitation wavelength of 494 nm and an emission wavelength of 516 nm by using a Pherastar fluorescence spectrophotometer. Calculations were carried out in Excel.

### Measurement of 8-HNT compound uptake.

Mid-log-phase L. donovani promastigotes were treated with and without compound 1 (5 μM) for 30 min at 28°C or 4°C. Triplicate samples of the culture (2 × 10^7^ cells; 1 ml) were harvested by centrifugation at 16,000 × *g* for 5 min. The supernatant (200 μl) was collected from each centrifuged sample, transferred to a fresh Eppendorf tube, and terminated by the addition of 400 μl acetonitrile (supernatant fraction). The remaining supernatant was discarded, and acetonitrile (60 μl) was added to each pellet fraction. Finally, 400 μl acetonitrile was added to 200 μl of culture (equivalent of 4 × 10^6^ cells) (total fraction). All fractions were then centrifuged at 16,000 × *g* for 10 min and diluted as required with MilliQ water. All samples were analyzed in triplicate on a Waters Acquity UPLC instrument coupled to a Waters micro TQs mass spectrometer. Chromatographic separation was achieved by using a Waters BEH (bridged ethylene hybrid) C_18_ column (1.7-μm particle size, 2.1 by 50 mm), with a flow rate of 0.6 ml min^−1^, an injection volume of 0.2 μl, and the following 2-min gradient: 95% mobile phase A initially, with holding for 0.3 min before decreasing to 5% mobile phase A over the next minute and holding for a further 0.49 min before returning to 95% mobile phase A. Mobile phase A was water plus 0.1% formic acid, and mobile phase B was methanol plus 0.1% formic acid. Cone voltage, collision energy, and MRM (multiple reaction monitoring) transition were optimized at 50 V, 28 V, and 367.06 > 124.92 (parent ion transition to daughter ion), respectively, using the automatic QuanOptimize feature. Additional MS parameters were as follows: positive electrospray ionization, capillary voltage of 40 V, desolvation temperature of 500°C, source temperature of 150°C, desolvation gas (nitrogen) at 1,000 liters/h, and cone gas (nitrogen) at 15 liters/h.

### Fluorescence-based assay to determine the binding of zinc by 8-HNT compounds.

ZnCl_2_ (5 μM) was incubated with various concentrations of our three 8-HNT compounds followed by the addition of 125 nM FluoZin-3 salt dye. Reaction mixtures were preincubated for 20 min at RT and then aliquoted into a black 96-well plate. Fluorescence was then measured with a Pherastar spectrophotometer (excitation at 485 nm and emission at 520 nm). Calculations were carried out in Excel.

### Spectrophotometric assay to determine the stoichiometry for 8-HNT compounds binding to divalent cations.

UV-visible (UV-Vis) titrations were carried out essentially as described previously (65, 66). Aliquots of metal divalent cations (concentrated stocks were made initially in water and then diluted in methanol) were added to a fixed concentration of 50 μM 8-HNT compound in methanol. After each aliquot addition, the incubation mixture was left to stabilize for 5 min at 25°C prior to scanning. Wavelength scanning was carried out at between 260 and 510 nm by using a UV-2401 PC UV-Vis spectrophotometer (Shimadzu). For Job's plot analyses (66), the combined concentration of 8-HNT and cation was fixed at 200 μM, and only the molar ratio of cation versus 8-HNT compound varied. Changes of the absorption at 475, 390, and 375 nm were indicative of cation complex formation with compounds 1 to 3, respectively. Specific absorbance at these wavelengths was determined and plotted against the molar ratio of compound to cation to determine complex stoichiometry (66).

### Accession number(s).

The complete RIT-seq data sets reported in this paper have been deposited in the European Nucleotide Archive (accession numbers PRJEB21478, PRJEB21479, and PRJEB21480 for compounds 1 to 3, respectively). All other relevant data are within this paper and the supplemental material.

## Supplementary Material

Supplemental file 1

Supplemental file 2
