# Supplementary material for: Antitrypanosomal 8-Hydroxy-Naphthyridines Are Chelators of Divalent Transition Metals
Source: Antimicrob Agents Chemother. 2018 Jul 27;62(8):e00235-18. doi: 10.1128/AAC.00235-18 (PMC6105827; doi:10.1128/AAC.00235-18)
Supplement: Supplemental file 1 [file zac008187328s1.pdf]

Anti-trypanosomal 8-hydroxy-naphthyridines are chelators of divalent transition metals

Richard J. Wall[1], Sonia Moniz[1], Michael G. Thomas[1], Suzanne Norval[1], Eun-Jung Ko[1], Maria Marco[2], Timothy J. Miles[2], Ian H. Gilbert[1], David Horn[1], Alan H. Fairlamb[1] and Susan Wyllie[1,#]

[1] The Wellcome Trust Centre for Anti-Infectives Research, Division of Biological Chemistry and Drug Discovery, School of Life Sciences, University of Dundee, Dundee, UK.

[2]Diseases of the Developing World, GlaxoSmithKline, Calle Severo Ochoa 2, 28760, Tres Cantos, Madrid, Spain.

Running title: *Anti-trypanosomal divalent cation chelators*

#To whom correspondence should be addressed: Dr. Susan Wyllie, The Wellcome Trust Centre for Anti-Infectives Research, Division of Biological Chemistry and Drug Discovery, School of Life Sciences, University of Dundee, Dundee, UK. Telephone: (+44)1382 38 5761; E-mail: s.wyllie@dundee.ac.uk

Supplementary figures

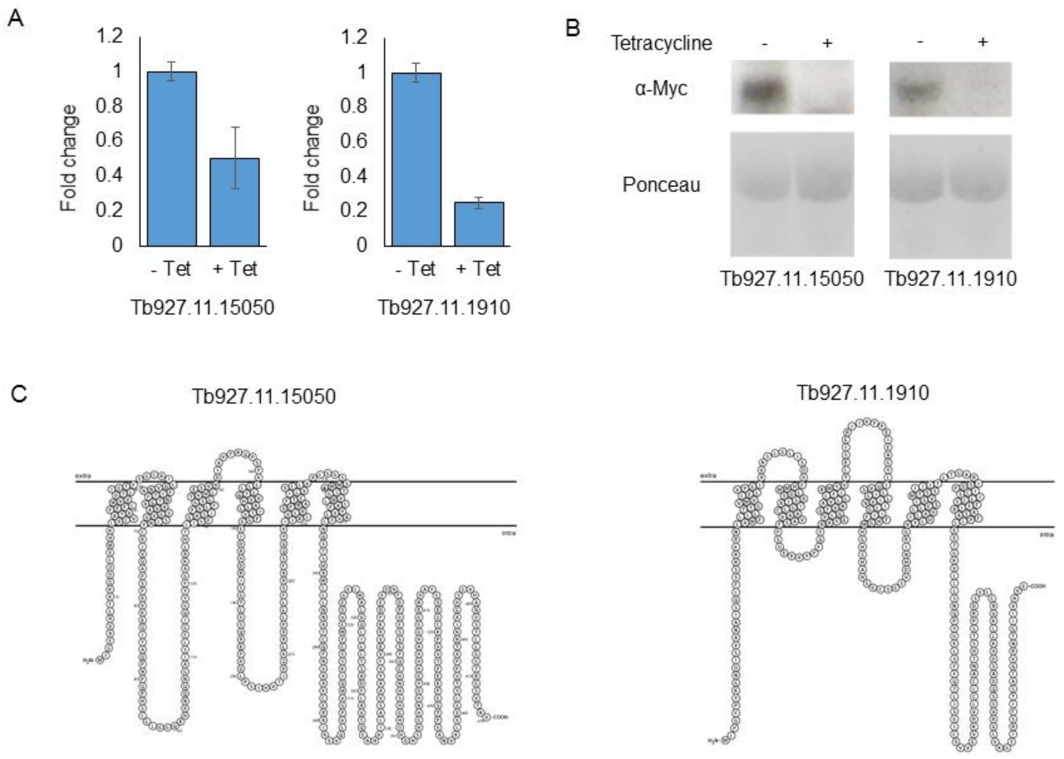

Figure S1. Structural characterisation and validation of major "hits" from RIT-seq screens of 8-HNT compounds. *A*, Quantitative RT-PCR confirming downregulation of the Tb927.11.1910 and Tb927.11.15050 transcript levels in stem-loop RNAi cell lines following the addition of tetracycline. *B*, Western blot confirming the RNAi-induced knockdown of the endogenously 12 x MYC-tagged proteins encoded by Tb927.11.1910 and Tb927.11.15050 in the presence of tetracycline. Blots were probed with a mouse monoclonal α-MYC antibody (1:500 dilution) followed by rabbit (anti-mouse IgG) – HRP-tagged antiserum (1:10,000 dilution). Ponceau S staining of membranes prior to western analysis acts as a loading control. *C*, A prediction of potential transmembrane domains within the proteins encoded by genes Tb927.11.1910 and Tb927.11.15050. This analysis suggests that both proteins maintain 6 transmembrane spanning domains.

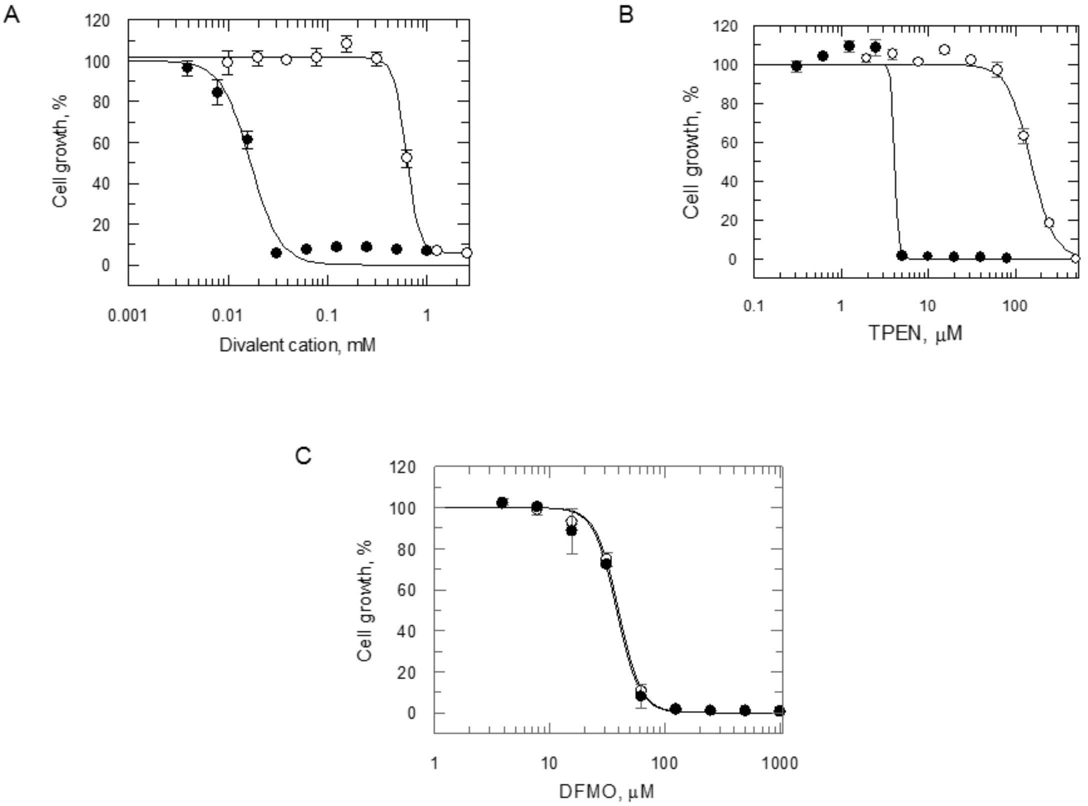

Figure S2. Control EC_{50} determination against bloodstream-form *T. brucei*. *A*, Dose response curves for $FeCl_2$ and $ZnCl_2$ against *T. brucei*. EC_{50} values for $FeCl_2$ (●) and $ZnCl_2$ (○) were 16.8 ± 1.4 µM and 619 ± 12.6 µM, respectively. *B*, Dose response curves for *T. brucei* in the presence of the Zn^{2+} chelator TPEN in the presence and absence of $ZnCl_2$ (200 µM). EC_{50} values for TPEN (●) and TPEN in the presence of $ZnCl_2$ (○) were 4.1 ± 0.9 and 150.7 ± 6.7 µM, respectively. All values represent the mean ± SEM for at least 3 experiments. *C*, Dose response curves for DFMO in the presence and absence of $ZnCl_2$ (200 µM) against *T. brucei.* EC_{50} values for DFMO (●) and DFMO in the presence of $ZnCl_2$ (○) were 39.5 ± 0.9 µM and 38.1 ± 1.5 µM, respectively.

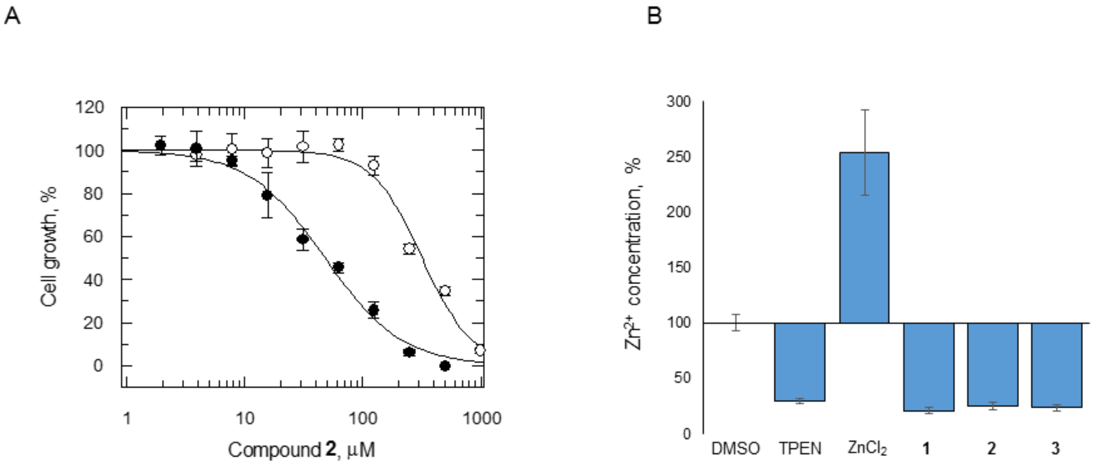

Figure S3. 8-HNT compounds act as chelators in human HepG2 cells. *A*, Dose response curves of compound **2** in the presence (○) and absence (●) of 100 µM $ZnCl_2$ against human HepG2 cells. In the absence of exogenous $ZnCl_2$ the EC_{50} value of compound **2** was 47.8 ± 4.0 µM while in presence of divalent cation the EC_{50} value shifted to 318.9 ± 4.0 µM. EC_{50} values represent the mean ± SEM for at least 3 experiments. *B*, Intracellular measurement of zinc as a percentage in HepG2 cells normalised to DMSO control (100%). Levels of intracellular zinc were measured using the fluorescent zinc reporter FluoZin-3 (final concentration - 5 µM), as described in the methods and in the presence of the zinc chelator TPEN (10 µM), exogenous $ZnCl_2$ (100 µM) or compounds **1-3** (100 µM). All values represent the mean ± SEM for at least 3 experiments.

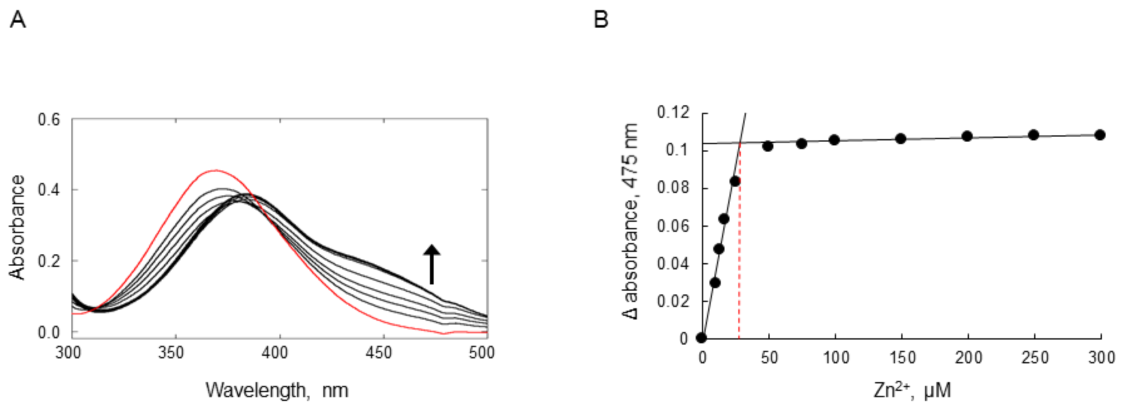

Figure S4. Characterisation of Zn^{2+} binding to compound 1. *A*, Family of UV-vis spectra taken in the course of titration of **1** (50 µM in methanol) with Zn^{2+}. The spectrum of compound **1** in the absence of Zn^{2+} is shown as a solid red line and the solid black lines represent compound **1** in the presence of increasing concentration of Zn^{2+}. Please note the Zn^{2+}–dependent increase of absorbance at 475 nm (highlighted by a black arrow). *B*, Plot of change in absorbance at 475 nm relative to increasing concentrations of Zn^{2+}. Dashed red line represent the extrapolated concentration of Zn^{2+} required to saturate binding to 50 µM compound **1**.

Supplementary tables S1-3. Hits identified from RITseq screening of three 8-HNT compounds. Hit list showing RPKM for barcoded reads (×10^3); only genes with RPKM >1000 for barcoded reads are included.

Supplementary table S4: EC_{50} values for 8-HNT compounds against *L. donovani* in the presence and absence of divalent cations.

Divalent cation	EC_{50} values, µM								
	Compound **1**			Compound **2**			Compound **3**		
	WT	Plus cation	Shift	WT	Plus cation	Shift	WT	Plus cation	Shift
$ZnCl_2$	0.8 ± 0.01	8.3 ± 0.3	11.0	0.2 ± 0.04	46.8 ± 2.1	265.7	0.2 ± 0.01	26.8 ± 0.9	120.1
$FeCl_2$	0.8 ± 0.03	1.6 ± 0.05	2.0	0.1 ± 0.01	0.9 ± 0.03	6.8	0.1 ± 0.004	0.3 ± 0.01	1.9

EC_{50} values represent the mean ± SD for at least 3 experiments.

Supplementary table S5: Primers used in study.

Name	Sequence (5' - 3')	Notes
HL1FW	GATCGGGCCCGGTACCACTAGTGCGGCAACGTTCTT	ApaI
HL1RV	GATCTCTAGAGGATCCCAGCGGCTCTGTAAAACACA	XbaI
HL2FW	GATCGGGCCCGGTACCTGTCACTGAACTACTGCCGC	ApaI
HL2RV	GATCTCTAGAGGATCCGATGCACGTGAAAGCAGAAA	XbaI
HL3FW	GATCGGGCCCGGTACCGGAGGTATCCGTTTGGGTATG	ApaI
HL3RV	GATCTCTAGAGGATCCCACCAACAACACTGCATATCTC	XbaI
Seq1	AATAGTGGACTCTTGTTCCA	
Seq2	AAAGGGGGATGTGCTGCAAG	
Myc1FW	GATCAAGCTTGTACATGCCCTGCTTATTACTGT	HindIII
Myc1RV	GATCTCTAGAAACTGCGGTGACGGAACCC	XbaI
Myc2FW	GATCAAGCTTGCTATTGTTTCTGGCACTGAGC	HindIII
Myc2RV	GATCTCTAGATTCTACATGAACAATAATTCCGGTATTC	XbaI
IntMyc1	CGACATAGCACACACGCGAC	
IntMyc2	CCATGTCCAAGGAACAGGTTG	
MycRev	GGCAGGTCTTCTTCAGAGATC	
HL1qPCRFW	GAGCAGCTGTGTGAAGTTGTTG	
HL1qPCRRV	CGGCGAGACTAATGGCTTCC	
HL2qPCRFW	GTCAGAAAGCCTGATGAAGAAGA	
HL2qPCRRV	CATAGTCATGATTGTGTCCATGAG	
HL3qPCRFW	GAGATATGCAGTGTTGTTGGTGTT	
HL3qPCRRV	CTGATCAACCGAATCGTGCTTC	
tert FW	GAGCGTGTGACTTCCGAAGG,	
tert RV	AGGAACTGTCACGGAGTTTGC	

Restriction endonuclease sites are underlined.
